# The response to prolonged fasting in hypothalamic serotonin transporter availability is blunted in obesity

**DOI:** 10.1016/j.metabol.2021.154839

**Published:** 2021-07-29

**Authors:** Katy A. van Galen, Jan Booij, Anouk Schrantee, Sofie M. Adriaanse, Unga A. Unmehopa, Eric Fliers, Gary J. Schwartz, Ralph J. DiLeone, Kasper W. ter Horst, Susanne E. la Fleur, Mireille J. Serlie

**Affiliations:** a Department of Endocrinology and Metabolism, Amsterdam University Medical Centers, Location AMC, Amsterdam, the Netherlands; b Department of Radiology and Nuclear Medicine, Amsterdam University Medical Centers, Location AMC, Amsterdam, the Netherlands; c Fleischer Institute for Diabetes and Metabolism, Department of Medicine, Albert Einstein College of Medicine, Bronx, NY, USA; d Department of Psychiatry, Yale University School of Medicine, New Haven, CT, USA; e Laboratory of Endocrinology, Department of Clinical Chemistry, Amsterdam Neuroscience, Amsterdam University Medical Centers, University of Amsterdam, the Netherlands; f Netherlands Institute for Neuroscience, An Institute of the Royal Netherlands Academy of Arts and Sciences (KNAW), Amsterdam, the Netherlands

**Keywords:** Obesity, Food intake, Fasting, Serotonin, Dopamine, SPECT

## Abstract

**Background and aims:**

Serotonergic and dopaminergic systems in the brain are essential for homeostatic and reward-associated regulation of food intake and systemic energy metabolism. It is largely unknown how fasting influences these systems or if such effects are altered in humans with obesity. We therefore aimed to evaluate the effects of fasting on hypothalamic/thalamic serotonin transporter (SERT) and striatal dopamine transporter (DAT) availability in lean subjects and subjects with obesity.

**Methods:**

In this randomized controlled cross-over trial, we assessed the effects of 12 vs 24 h of fasting on SERT and DAT availability in the hypothalamus/thalamus and striatum, respectively, using SPECT imaging in 10 lean men and 10 men with obesity.

**Results:**

As compared with the 12-h fast, a 24-h fast increased hypothalamic SERT availability in lean men, but not in men with obesity. We observed high inter-individual variation in the effects of fasting on thalamic SERT and striatal DAT, with no differences between lean men and those with obesity. In all subjects, fasting-induced increases in circulating free fatty acid (FFA) concentrations were associated with an increase in hypothalamic SERT availability and a decrease in striatal DAT availability. Multiple regression analysis showed that changes in plasma insulin and FFAs together accounted for 44% of the observed variation in striatal DAT availability.

**Conclusion:**

Lean men respond to prolonged fasting by increasing hypothalamic SERT availability, whereas this response is absent in men with obesity. Inter-individual differences in the adaptations of the cerebral serotonergic and dopaminergic systems to fasting may, in part, be explained by changes in peripheral metabolic signals of fasting, including FFAs and insulin.

## 1. Introduction

Obesity is defined as an excess of body fat and develops when long-term energy consumption exceeds energy expenditure [1]. Over the last decades, the increased availability of affordable, energy-dense food has paralleled an increase in average food consumption in many countries, and this is believed to be the main driver of the current obesity pandemic [2,3]. On an individual level, the brain regulates many aspects of eating behavior and energy balance. Disturbances in the central regulation of food intake are hypothesized to underlie a pattern of eating behaviors in individuals with obesity that is characterized by ongoing caloric intake despite systemic energy surplus [4].

Two important systems in the regulation of eating behavior by the brain are the homeostatic system and the reward system. The homeostatic system’s primary function is to maintain energy homeostasis by matching the amount of calories consumed to the amount of calories expended [5]. The hypothalamus and brainstem are key brain regions for the homeostatic regulation of food intake [6,7]. In contrast, the reward system’s primary function is to integrate the motivational and rewarding aspects of eating behavior [8]; a key brain region is the striatum [9]. The homeostatic and reward systems are strongly interconnected, and there are strong anatomical and functional interactions between these regulatory systems [10,11]. The neurotransmitters serotonin and dopamine play important roles in the homeostatic and reward regulation [12,13], respectively; however, we note that many other neurotransmitters are involved [5,14]. Serotonin transporters (SERTs) and dopamine transporters (DATs) facilitate the reuptake of serotonin and dopamine from the synaptic cleft and thereby regulate intrasynaptic neurotransmitter levels [15,16].

Changes in serotonergic and/or dopaminergic signaling are hypothesized to contribute to obesity [17,18]. Reduced postprandial serotonergic signaling results in insufficient inhibition of additional food intake (i.e., beyond homeostatic needs) [19,20]. In turn, postprandial striatal dopamine release is blunted in diet-induced obese mice, suggesting a reduction in the rewarding aspects of food intake; this may promote a compensational increase in food intake [21]. Although methods to directly measure cerebral serotonin and dopamine concentrations in humans in vivo are unavailable, molecular neuroimaging techniques do provide surrogate markers for the assessment of these neurochemicals in humans. For instance, we have previously observed that SERT availability in the thalamus, a region that connects the homeostatic and rewards systems, is dependent on the timing of food intake during a hypocaloric diet intervention [22]. Other studies that have used molecular neuroimaging to measure cerebral serotonergic and dopaminergic signaling also support a role for serotonin and dopamine signaling in human obesity [23]. Taken together, the data suggest that obesity is associated with decreased tonic serotonin levels, with increased tonic dopamine levels, and with reduced phasic dopamine release in the synapse [23]. However, these studies were conducted in subjects who had differential basal metabolic states at the time of imaging. This is important, because the results of animal studies suggested effects of fasting duration on the serotonergic and dopaminergic neurotransmitter systems [24–26]; differences in fasting duration may thus have affected the outcomes of previous human studies.

From an evolutionary perspective, the metabolic and behavioral responses to foods are essential to survive periods of food scarcity [27]. Among the responses, the metabolic adaptation to fasting requires a transition from glucose to lipid as the primary energy substrate. In line, the response to fasting is characterized by decreased plasma insulin, glucose, and leptin levels and by increased plasma free fatty acid (FFA), glucagon, ketone body, and ghrelin levels [27–29]. Data from animal studies suggest that changes in these peripheral hormone and nutrient levels affect the central serotonergic and dopaminergic systems [26,30–34], thus signaling to the brain when systemic nutrient availability is low. In this regard, obesity has been associated with metabolic inflexibility (i.e., a diminished capacity for mitochondrial fuel switching and an impaired regulation of mitochondrial fuel balance [35]), a condition that may become especially apparent during prolonged fasting. In the context of metabolic inflexibility, changes in peripheral hormone and nutrient levels may thus affect how the homeostatic circuitry of an individual with obesity adapts to prolonged fasting and/or other nutritional states. Finally, the homeostatic system directly interacts with the reward system to enhance the motivational and rewarding aspects of food intake under fasting conditions, thereby contributing to an integrated behavioral response that increases the likelihood of short-term restoration of energy balance [4,36]. It thus seems that fasting affects both the homeostatic and reward systems, and studying the metabolic and central adaptations to fasting in humans with normal weight or obesity will likely provide novel insights into the central serotonergic and dopaminergic systems, the regulation of eating behavior, and the pathogenesis of obesity.

Here, we aimed to: i) assess the effects of fasting on central serotonin and dopamine transporter availability, ii) determine differences in the effects of fasting between lean humans vs humans with obesity, and iii) explore the relationships between fasting-induced changes in peripheral metabolic signals, energy substrate oxidation and the central serotonergic and dopaminergic systems.

## 2. Methods

### 2.1. Design

This randomized controlled crossover study was designed to study the effects of 12 vs 24 h of fasting on hypothalamic and thalamic SERT availability, striatal DAT availability, and parameters of systemic metabolism in lean subjects and subjects with obesity. The protocol was approved by the Academic Medical Center medical ethics committee. All subjects provided written informed consent in accordance with the Declaration of Helsinki. The study was prospectively registered in the Netherlands Trial Registry (http://www.trialregister.nl: NTR6609).

### 2.2. Subjects

We recruited 10 lean subjects and 10 subjects with obesity from the general population in the Amsterdam metropolitan area via local advertisements. Subjects were eligible to participate if they: i) were men, ii) were aged 50–75 years (to prevent radiation exposure in younger subjects [37]), iii) had either a body mass index (BMI) <25 kg/m^2^ (lean subjects) or BMI >30 kg/m^2^ (subjects with obesity), and iv) had stable weight (<5% weight change) for at least 3 months prior to the study assessments. Exclusion criteria were: i) use of any medication (except for thyroid hormone, antihypertensive, and/or lipid-lowering drugs); ii) any somatic disorder (except for adequately treated hypothyroidism, hypertension, and/or dyslipidemia); iii) history of any psychiatric or eating disorder; iv) shift work; v) irregular sleeping habits; vi) regular vigorous exercise (>3 h/week); vii) restrained eaters [38]; viii) substance abuse (smoking, alcohol >3 units/day, and/or recreational drugs); or ix) any contra-indication for magnetic resonance imaging (MRI). All subjects completed a medical evaluation, including history, physical examination, and blood tests. Systemic insulin sensitivity was estimated using the fasting plasma insulin concentration, where fasting insulin >74 pmol/L indicates insulin resistance [39].

### 2.3. Randomization and fasting protocols

We used a randomized controlled crossover design to compare the effects of 12 vs 24 h of fasting. All subjects underwent two study days: one after a 12-h overnight fast, and one after a 24-h fast that started at 10–12 AM on the day before neuroimaging. Subjects received a sealed envelope, containing instructions to fast for 12 h before the first study day and 24 h before the second study day, or vice versa. Allocation to treatment order was randomized and stratified (1:1) by treatment and by obesity using the GraphPad QuickCalcs website (http://graphpad.com/quickcalcs/randomize2/). The primary investigator was blinded to allocation.

**Fig. 1.**
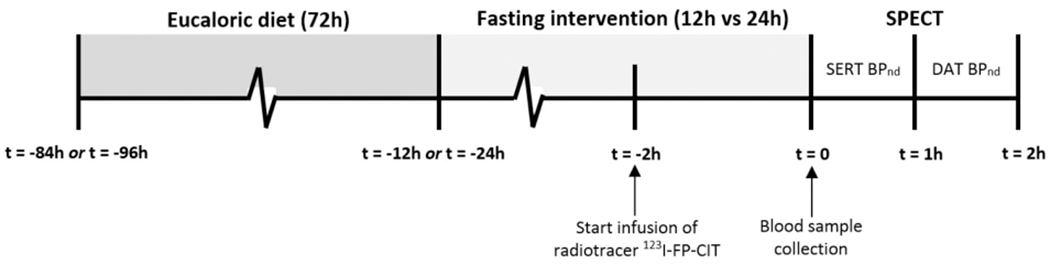
Timeline of study protocol.

We defined fasting as abstinence from all foods and drinks, except for tap water. Subjects started their fast exactly 12 or 24 h before the first brain scan (Fig. 1). To limit confounding due to different dietary habits, subjects were instructed to maintain an eucaloric diet for 72 h before starting their fast. The eucaloric diet was identical before both fasting interventions. It was created individually on the basis of resting energy expenditure (REE), as measured by indirect calorimetry (Vmax Encore 29, Carefusion, San Diego, CA, USA) and estimated physical activity. The diet consisted of three meals and two snacks per day, and had a standardized macronutrient ratio of 50% carbohydrates, 30% fat, and 20% proteins.

### 2.4. Brain single-photon emission computed tomography (SPECT) imaging

Hypothalamic and thalamic SERT and striatal DAT availability were assessed using SPECT imaging and the radiotracer ^123^I-*N*ω-fluoropropyl-2β-carboxymethoxy-3β-(4-iodophenyl)nortropane (^123^I-FP-CIT), a method that we and others have previously validated [40–44]. SERTs and DATs facilitate the synaptic reuptake of serotonin and dopamine, respectively, into the pre-synaptic neuron and thereby regulate the amounts of neurotransmitter available for signaling [45]. In vivo-assessed SERT and DAT availability reflect a combination of the expression of the transporter (B_max_) and the affinity of the radiotracer for the given transporter (1/K_d_ [46]). In other words, an increase in B_max_ and a decrease in K_d_ can both increase ^123^I-FP-CIT binding (B_max_/K_d_). The K_d_ can thus be influenced by changing the synaptic levels of neurotransmitters or a change in the conformational state of the transporter [47]. An increase in transporter availability (i.e., higher binding of ^123^I-FP-CIT) reflects higher transporter expression (i.e., higher B_max_) or higher affinity (i.e., lower K_d_) induced by a decrease in the concentration of endogenous neurotransmitter, a conformational change, or a combination of these factors. Because ^123^I-FP-CIT binding in the hypothalamus/thalamus and the striatum can be blocked in vivo by selective SERT and DAT reuptake inhibitors, respectively, binding of ^123^I-FP-CIT in the hypothalamus/thalamus and the striatum predominantly reflect SERT and DAT, respectively [40,41]. We have previously demonstrated that hypothalamic/thalamic SERT and striatal DAT availability can be optimally imaged 2 and 3 h after the administration of the radiotracer, respectively [43,48].

Subjects were administered an intravenous bolus of approximately 100 MBq ^123^I-FP-CIT (specific activity >750 MBq/nmol; radiochemical purity >98%; produced in accordance with GMP guidelines; GE Healthcare, Eindhoven, The Netherlands). Next, SPECT imaging was performed using the InSPira HD system, a brain-dedicated SPECT camera (Neurologica, Boston, USA) with the following parameters: acquisition time per slice, 180 s; slice thickness, 4 mm. Slices were acquired from the level of the cerebellum up to the striatum. SPECT images were reconstructed with an iterative expectation maximization algorithm and corrected for attenuation by manually aligning an adult head template [41,49]. To limit thyroid uptake of free radioactive iodide, all subjects were pre-treated with potassium iodide.

### 2.5. Brain MRI scanning

**Fig. 2.**
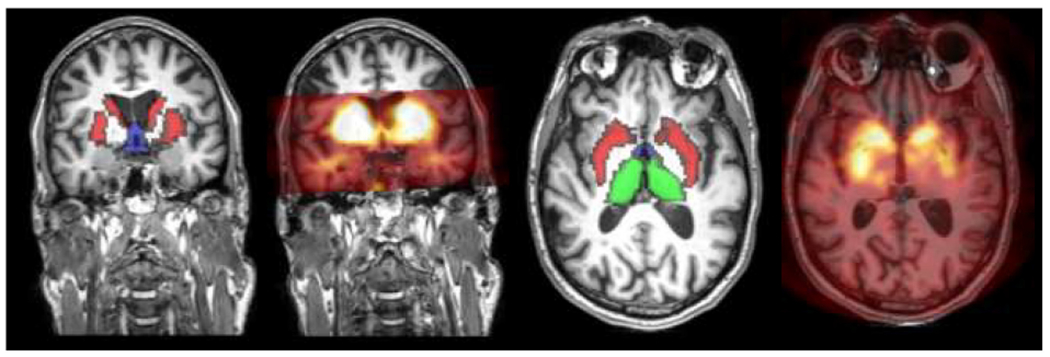
Representative T1w anatomical brain MRI scans overlaid with co-registered SPECT images and ROI masks. Striatal (red mask), hypothalamic (blue mask), and thalamic (green mask) uptake of the radiotracer 123I-FP-CIT.

For anatomical reference, a T1-weighted (T1w) MRI scan of the brain was obtained on a 3.0-T Ingenia MR scanner (Philips Healthcare, Best, The Netherlands) using a 32-channel receive-only head coil and the following scan parameters: TR/TE 7/3.18 ms; flip angle 9°; 1 mm isotropic resolution. Individual SPECT scans were co-registered to individual MRI scans using 6-parameter rigid body registration in SPM12 (Wellcome Centre for Neuroimaging, London, UK) (Fig. 2). To optimize registration, a high-intensity striatal mask was superimposed on the T1w scan to resemble the contrast on the SPECT scan.

### 2.6. Region-of-interest (ROI) analysis

Hypothalamic and thalamic SERT as well as striatal DAT availability were quantified using ROI analysis. We used Freesurfer version 5.3.0 [50,51] to obtain thalamic and striatal masks from individual T1w MRI scans. Hypothalamic masks were outlined manually using anatomical landmarks on ITK-SNAP version 3.4.0 [40,52,53]. The cerebellum was used to quantify nonspecific radiotracer activity; ^123^I-FP-CIT activity in this region does not reflect binding to either SERTs or DATs, since the cerebellum is almost devoid of these transporters [41,54,55]. We obtained cerebellar masks by warping the cerebellum (without vermis) from the Harvard-Oxford subcortical atlas (https://fsl.fmrib.ox.ac.uk/fsl/fslwiki/Atlases) to individual T1w MRI using FSL (FMRIB Software Library, version 5.0.8, Oxford, UK). For each ROI, we measured the availability of regional SERT and DAT [i.e., binding potential (BP_ND_) of ^123^I-FP-CIT] by calculating the specific-to-nonspecific binding ratio, using the following formula: BP_ND_ = (mean hypothalamic, thalamic or striatal binding – mean cerebellar binding)/mean cerebellar binding. Fasting-induced changes in SERT and DAT availability were defined as BP_ND_ after a 24-h fast, relative to BP_ND_ after a 12-h fast.

### 2.7. Metabolic effects of fasting

Venous blood samples were drawn after both fasting interventions to determine the effect of a 12-h vs 24-h fast on circulating hormone and nutrient levels. In addition, indirect calorimetry was performed to measure REE and the respiratory quotient (RQ) after 12 vs 24 h of fasting.

### 2.8. Laboratory analysis

Plasma glucose was determined with the glucose oxidase method using a Biosen C-line plus glucose analyzer (EKF Diagnostics, Barleben/Magdeburg, Germany). Plasma insulin was determined by immunoassay on an Atellica system (Siemens, Erlangen, Germany) with intra-assay variation of 3% and inter-assay variation of 7%. Plasma FFAs were determined by an enzymatic colorimetric method (NEFA C test kit; Wako Chemicals, Neuss, Germany) with intra-assay variation of 1% and inter-assay variation of 4–15%. Plasma glucagon was determined by radioimmunoassay (Linco Research, St Charles, MO, USA) with intra-assay variation of 4–8% and inter-assay variation of 6–11%. Plasma leptin was determined by radioimmunoassay (Millipore, Burlington, MA, USA) with intra-assay and inter-assay variation of 6%. Plasma ghrelin was determined by radioimmunoassay (Millipore, Burlington, MA, USA) with intra-assay variation of 4% and inter-assay variation of 6%.

### 2.9. Statistical analysis

Data are presented as mean ± standard deviation (SD) or median [interquartile range (IQR)], unless stated otherwise. Data were assessed for normality by inspection of histograms; outliers were detected using Grubbs tests (upper 2.5% significance level). Outcomes were evaluated using a repeated-measures analysis of variance (ANOVA), with fasting duration as the within-subjects factor and group (lean men vs men with obesity) as the between-subjects factor. Differences between groups were evaluated using *t*-tests or Mann-Whitney *U* tests. Fasting-induced changes within groups were assessed using paired t-tests or Wilcoxon signed-rank tests. Correlations were evaluated using Pearson’s or Spearman’s coefficient. Multiple linear regression analysis was performed to assess the independent associations between peripheral fasting signals and regional SERT or DAT availability. Findings were considered significant if p < 0.05. SPSS version 26.0 (IBM, Armonk, NY, USA) was used for all statistical testing.

## 3. Results

### 3.1. Participants

Ten lean men and nine men with obesity completed the study (Table 1). One individual with obesity was excluded, because his SPECT scans could not be analyzed due to technical issues. Groups did not differ in age. Subjects with obesity had higher fasting plasma insulin concentrations, indicating insulin resistance [39], and higher fasting leptin concentrations. There were no differences between groups in hypothalamic and thalamic SERT and striatal DAT availability after the 12-h fast.

### 3.2. Metabolic effects of fasting

The 24-h fast had significant effects on parameters of systemic metabolism (Table 2), indicating ongoing metabolic adaptations when fasting duration is extended from 12 to 24 h. As compared with the 12-h fast, the 24-h fast induced reductions in plasma glucose and leptin concentrations in both groups. In lean men, plasma insulin concentrations and the RQ also decreased, with the latter indicating a shift from carbohydrate towards lipid oxidation. In subjects with obesity, plasma insulin concentrations and the RQ did not decrease after 24, as compared with 12, hours of fasting, which is consistent with the hypothesis that obesity is associated with metabolic inflexibility [56]. In neither group, REE nor plasma FFA, glucagon, and ghrelin levels differed between the fasting durations. There were no between-group differences in the effects of prolonged fasting on fasting metabolites or hormones. As compared to the men with obesity, lean men tended to show a stronger effect of prolonged fasting on REE and RQ.

### 3.3. Effects of fasting on central SERT and DAT availability

**Fig. 3.**
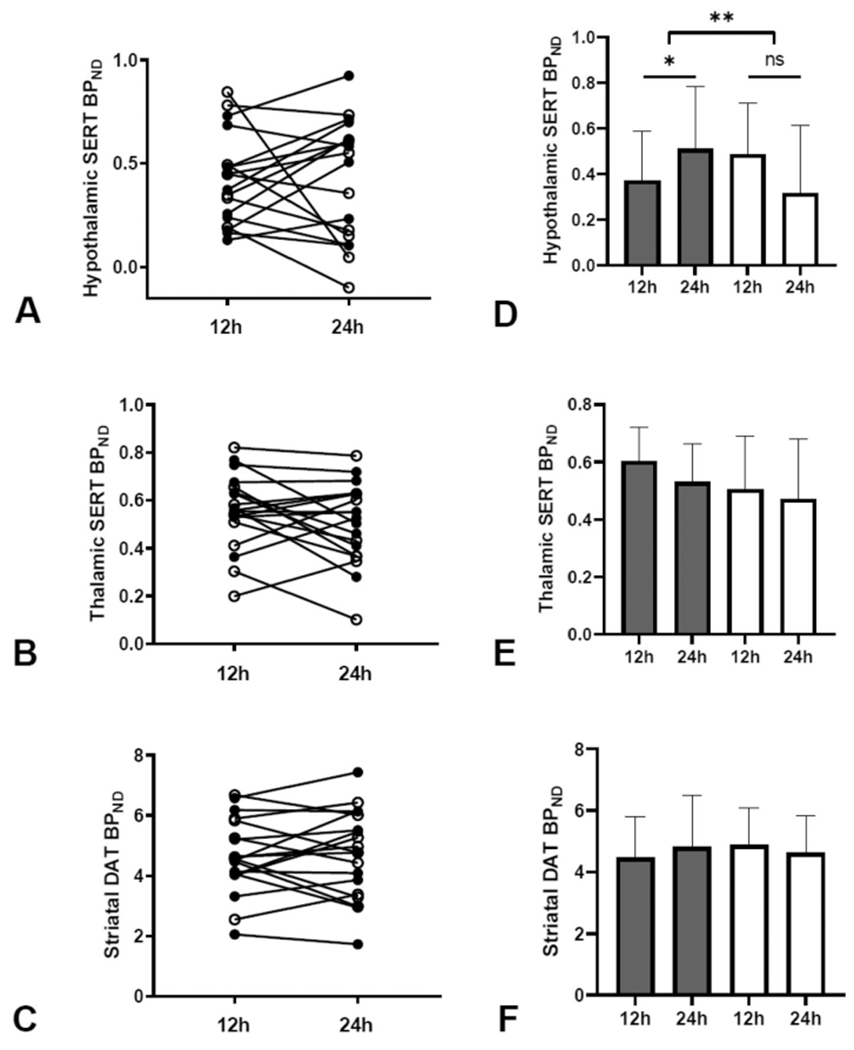
Radiotracer binding after a 12-h vs 24-h fast in lean men and men with obesity. (A) Hypothalamic SERT availability. (B) Thalamic SERT availability. (C) Striatal DAT availability. Mean radiotracer binding after a 12-hand 24-h fast in lean men and men with obesity. (D) Hypothalamic SERT availability. (E) Thalamic SERT availability. (F) Striatal DAT availability. Data are individual subjects (A–C) and mean ± SD (D–F). One outlier was removed (A + D). *p < 0.05 for paired t-test **p < 0.05 for fasting-obesity interaction on repeated measures ANOVA.

To study the effects of fasting on central SERT and DAT availability, we performed SPECT scans after 12 vs 24 h of fasting. We observed no overall differences in hypothalamic and thalamic SERT or in striatal DAT availability after the 24-h fast, as compared with the 12-h fast (Fig. 3A–C). However, as can be appreciated from the individual subjects’ data (Fig. 3A–C), we did observe high inter-individual variation in the effects of fasting. In this regard, we observed an important interaction between obesity and fasting duration on hypothalamic SERT availability (F_1,16_ = 6.474, p = 0.022, Fig. 3D, Table 2), indicating that fasting duration differentially affects hypothalamic SERT availability in lean individuals vs individuals with obesity. In lean men, hypothalamic SERT availability significantly increased by 48.3% ± 76.4% (p = 0.044) upon 24 h of fasting, which is in contrast to the unchanged hypothalamic SERT availability (−36.3% ± 71.1%, p = 0.179) in men with obesity (Table 2). We observed no interactions between obesity and fasting on thalamic SERT (F_1,17_ = 0.265, p = 0.614, Fig. 3E) or striatal DAT availability (F_1,17_ = 1.989, p = 0.176, Fig. 3F), suggesting that the high inter-individual variation in the effects of fasting on thalamic SERT and striatal DAT availability is not explained by differences in body weight.

### 3.4. Predictors of central SERT and DAT availability in fasting humans

**Fig. 4.**
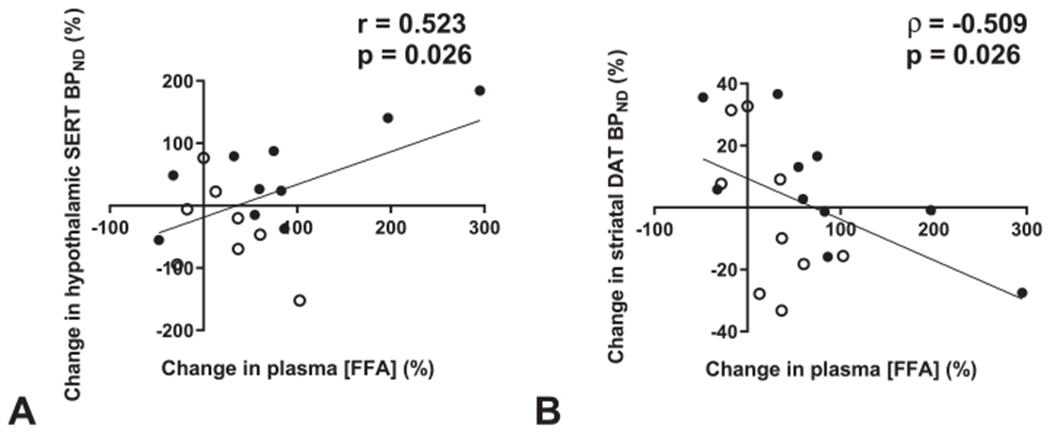
Fasting-induced changes in circulating FFAs predict central SERT and DAT availability upon prolonged fasting in humans. Scatterplots showing the relationship between fasting-induced changes in plasma FFA levels and (A) hypothalamic SERT availability or (B) striatal DAT availability in all subjects. Data are lean (•) subjects or subjects with obesity (∘) (A–B). One outlier was removed (A).

The metabolic adaptation to fasting includes changes in multiple plasma hormone and nutrient concentrations, all of which may signal nutritional depletion to the brain [26,30–34]. Therefore, we next explored if these changes might contribute to the observed inter-individual variation in transporter availability upon fasting. In all subjects, there were no correlations between fasting-induced changes in transporter availability and circulating glucose, insulin, glucagon, leptin, and ghrelin levels (not shown). Changes in plasma FFA levels correlated positively with changes in hypothalamic SERT (Fig. 4A) and negatively with changes in striatal DAT availability (Fig. 4B), indicating that a fasting-induced increase in plasma FFA levels was associated with an increase in hypothalamic SERT availability and with a decrease in striatal DAT availability. Plasma FFA levels did not correlate with thalamic SERT availability (r = 0.135, p = 0.580). Because increased plasma FFA levels reflect ongoing lipolysis and a crucial shift from carbohydrate to lipid metabolism during prolonged fasting, these data may indicate a role for circulating FFAs as a signal of systemic carbohydrate depletion to the central serotoninergic and dopaminergic systems.

### 3.5. Relation with insulin resistance

Individuals with peripheral insulin resistance also show blunted cerebral responses to insulin, suggesting central insulin resistance [57,58]. We hypothesized that normal insulin sensitivity is required to sense fasting-induced decreases in plasma insulin levels. To this end, we divided all subjects into an insulin-sensitive and an insulin-resistant group (Supplemental Table S1), using fasting plasma insulin concentrations as described [39]. In the 13 subjects with insulin sensitivity, changes in plasma insulin levels tended to negatively correlate with changes in striatal DAT availability (rho = −0.527, p = 0.064), suggesting that a decrease in plasma insulin levels was associated with an increase in striatal DAT availability. This was not observed in the six subjects with insulin resistance (rho = 0.143, p = 0.787). Changes in plasma insulin levels did not correlate with hypothalamic and thalamic SERT availability in the insulin-sensitive (rho = −0.154, p = 0.633 and rho = 0.071, p = 0.817, respectively) or insulin-resistant subjects (rho = −0.714, p = 0.111 and rho = −0.657, p = 0.156, respectively).

**Fig. 5.**
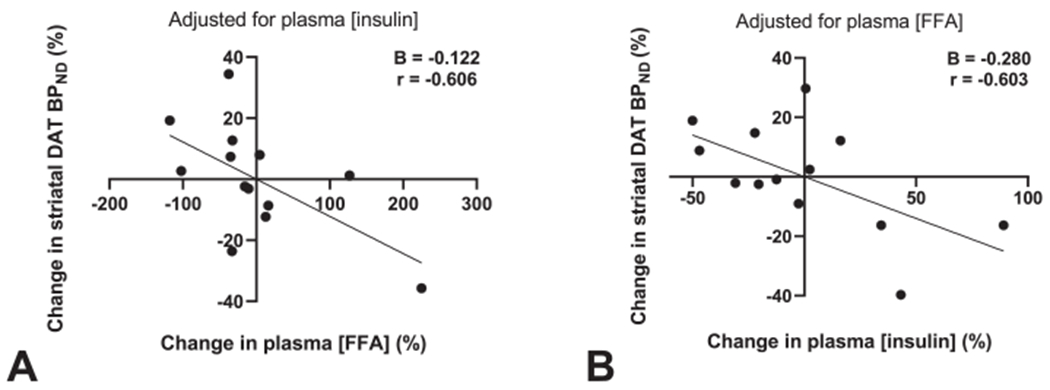
Fasting-induced changes in plasma FFA and insulin together accounted for 44% of the variation in striatal DAT availability (adj. R^2^ = 0.441, p = 0.022). Partial regression plots showing the relationship between fasting-induced changes in striatal DAT availability and (A) plasma FFA levels, after adjusting for plasma insulin levels or (B) plasma insulin levels, after adjusting for plasma FFA levels. Data represent the subset of insulin-sensitive subjects (n = 13).

Insulin inhibits cerebral FFA oxidation [59]; fasting-induced reductions in insulin signaling may thus increase neuronal FFA oxidation. We therefore hypothesized that changes in FFA and insulin levels together improve the accuracy of the prediction of changes in striatal DAT availability, at least in the subjects with insulin sensitivity. Indeed, multiple regression analysis showed that changes in plasma FFAs and insulin both significantly predicted changes in striatal DAT availability in insulin-sensitive subjects (Fig. 5A–B), together accounting for 44% of the observed variation (Supplemental Table S2). This finding is consistent with the concept that both low plasma insulin and high plasma FFAs may directly or indirectly provide a fasting signal to the central reward system.

## 4. Discussion

To the best of our knowledge, this is the first study to assess the effects of fasting on the central serotoninergic and dopaminergic systems in humans. Data from our study demonstrate that: i) a 24-h fast increases hypothalamic SERT availability in lean men, but not in men with obesity; ii) 24 h of fasting does not significantly affect mean thalamic SERT or striatal DAT availability in lean men nor in men with obesity, but with high inter-individual variation in the observed responses; and iii) fasting-induced changes in plasma FFA and insulin levels account for much of the observed variation.

Fasting differentially affected hypothalamic SERT availability in lean subjects vs subjects with obesity: in lean subjects, hypothalamic SERT availability increased upon the 24-h fast, whereas in individuals with obesity, hypothalamic SERT availability did not change significantly. An increase in hypothalamic SERT availability upon 24 h of fasting likely points to a decrease in hypothalamic serotonergic signaling: increased radiotracer binding either reflects increased synaptic SERT expression, which promotes reuptake of serotonin from the synapse, and/or it reflects increased radiotracer affinity for SERT induced by decreased synaptic serotonin levels [60,61]. In rodents and healthy humans, a short-term increase in serotonergic signaling reduces food intake, suggesting a role in satiety [62,63]. In contrast, a decrease in serotonergic signaling stimulates food-seeking behavior and intake in rodents [64–66]. Given the anorexigenic effects of hypothalamic serotonergic signaling, the fasting-induced increase in hypothalamic SERT availability in the lean healthy subjects likely reflects decreased serotonergic signaling, which is consistent with an adequate response to prolonged fasting that would contribute to the promotion of food-seeking behavior and restoration of energy balance [17]. In individuals with obesity, however, prolonged fasting did not change hypothalamic SERT availability. This may be explained by chronic perturbation in serotonergic signaling in obesity per se or by a difference in the overall metabolic response to 24 h of fasting [23,67].

We found that plasma FFA levels were the strongest predictors of hypothalamic SERT and striatal DAT availability. Circulating FFA levels increase during fasting due to the stimulation of adipose tissue lipolysis, and FFAs serve as an alternative fuel source [68]. Our data suggest that circulating FFAs may also function as a signal of nutritional depletion for the brain. In this regard, FFAs can enter the brain from the peripheral circulation, with plasma and brain concentrations of some FFAs showing direct correlations [69,70]. FFAs are natural ligands of free fatty acid receptors (FFARs), a group of orphan G-protein-coupled receptors (GPCRs) involved in the regulation of energy homeostasis [71]. Now, we demonstrate that a fasting-induced increase in plasma FFA levels is associated with an increase in hypothalamic SERT availability. Therefore, our data are consistent with the interpretation that FFAs are a direct signal of nutritional scarcity to the brain, contributing to behavioral adaptation through the central serotonergic system.

A fasting-induced increase in plasma FFA levels is also associated with a decrease in striatal DAT availability. In rodents, fasting decreases striatal DAT function [25,72], reducing the re-uptake of dopamine from the synaptic cleft. In line with this, DAT knock-out mice show increased motivation to obtain food [73]. In humans, reduced striatal DAT availability is associated with a faster response to a visual food stimulus, indicating enhanced attention for food [74]. Our present observations may suggest that a reduction in striatal DATs enhances the awareness for food-related stimuli, which, in turn, contributes to the increased likelihood of short-term food consumption. However, a role for hypothalamic/striatal FFA signaling in fasting-induced increases in the motivation for food may seem contrary to studies reporting an anorexigenic effect of intracerebroventricular infusions of select FFAs [75,76]. In rodents, insulin and glucose inhibit hypothalamic β-oxidation of FFAs, resulting in the cellular accumulation of FFAs and decreased food intake [59,77]. In the fasting state, however, systemic glucose and insulin levels are low, and the accumulation of FFAs is prevented by increased β-oxidation; this interaction may signal nutritional deprivation to the brain [59]. Taken together, we show that fasting-induced increases in plasma FFA levels are associated with changes in both the central serotonergic and dopaminergic systems that may increase the likelihood of short-term food intake.

Alongside changes in plasma FFA levels, changes in circulating insulin levels contributed to the prediction of fasting-induced changes of striatal DAT availability in insulin-sensitive subjects. In rodents, a growing body of literature supports a role for insulin signaling to the mesolimbic dopamine system in the adaption of motivational and rewarding aspects of food intake to homeostatic needs [78]. Interestingly, high-fat diet-induced insulin resistance abolished the effects of insulin on striatal dopamine release and reuptake, which could be restored with an insulin receptor sensitizing agent [79]. In humans, insulin receptor expression is high in mesolimbic brain regions [80]. Humans with systemic insulin resistance have been reported to show attenuated insulin-evoked brain responses [57]. Intact insulin sensitivity may be required to sense fasting-induced decreases in circulating insulin levels. Thus, our findings suggest that insulin may play a role in the adaptation of the dopaminergic system to fasting in insulin-sensitive humans.

Previous studies that have assessed the cerebral serotonergic and dopaminergic systems in humans have done so following different durations of fasting [23]. This complicates the interpretation of those studies, because it is unknown how fasting may have affected the systems under investigation. Here, we show that, the effects of 12 vs 24 h of fasting on hypothalamic SERT availability differ between lean men and men with obesity. In addition, although mean thalamic and striatal transporter availability did not differ after 12 vs 24 h of fasting, we did observe high inter-individual variation in transporter availability after 12 vs 24 h of fasting. In this regard, individual variation in fasting FFA and insulin levels accounted for much of the observed variation. However, given that parts of the variation in the effects of fasting remain unexplained, it seems fair to assume that unmeasured parameters of fasting influence the serotonergic and dopaminergic systems as well. For instance, an increase in ketone bodies may also signal ‘fasting’ to the brain [81]. We therefore recommend that investigators consider possible effects of fasting on the serotonergic and dopaminergic systems and standardize fasting duration prior to SPECT imaging in future studies.

This study was designed to study the effects of prolonged fasting on central SERT and DAT availability, and we acknowledge that our findings on the *predictors* of central SERT and DAT availability should be interpreted as secondary endpoints. We also note that some of our observations may have been influenced by unmeasured signals of fasting, including ketone bodies. In addition, we only studied men and our findings cannot be extrapolated to women or people in different age groups. Finally, most of the subjects with obesity were classified as class I obesity [1] and we cannot draw conclusion on individuals with more severe obesity. Going forward, it will be of interest to directly assess how FFAs and insulin signal to the hypothalamic serotoninergic and striatal dopaminergic systems, for instance during infusion or hyperinsulinemic clamp experiments. Finally, due to the limited spatial resolution of the dedicated brain tomographic SPECT system, it was not possible to examine subregional differences *within* the hypothalamus.

In conclusion, we show that prolonged fasting increases hypothalamic SERT availability in men with normal weight, but not in men with obesity. We observed no overall effects of fasting on thalamic SERT or striatal DAT availability, but fasting-induced responses in these regions showed high inter-individual variation. Fasting-induced increases in plasma FFAs were associated with increased hypothalamic SERT and decreased striatal DAT availability, and changes in circulating FFA and insulin concentrations together accounted for almost half of the dopaminergic response to fasting. Taken together, the data indicate that human obesity is associated with a blunted hypothalamic serotonergic response to fasting and that FFAs and insulin might serve as important peripheral messengers to the brain to relay information on the nutritional state.

## Supplementary Material

SUPPLEMENTARY TABLES S1 andS2

## Figures and Tables

**Table 1:** Characteristics of study participants after the 12-h fast (n = 19).

	Lean men (n = 10)	Men with obesity (n = 9)	p
Age (years)	64 ± 7	65 ± 8	0.828
Length (cm)	174 ± 9	177 ± 6	0.499
Weight (kg)	70 ± 11	102 ± 14	<0.001
BMI (kg/m^2^)	23.0 ± 1.8	32.6 ± 2.8	<0.001
Waist circumference (cm)	90 ± 9	117 ± 12	<0.001
REE (kcal/kg/day)	20.2 ± 3.8	18.6 ± 2.5	0.283
RQ	0.78 ± 0.07	0.77 ± 0.05	0.737
Glucose (mmol/L)	4.8 ± 0.4	5.5 ± 1.4	0.127
FFA (mmol/L)	0.48 [0.38–0.63]	0.45 [0.35–0.58]	0.653
Insulin (pmol/L)	28 [26–48]	130 [58–170]	0.002
Glucagon (ng/L)	87 [70–116]	88 [70–133]	0.595
Leptin (μg/L)	6 [5–9]	23 [16–33]	<0.001
Ghrelin (pg/mL)	33.8 [19.8–47.3]	13.7 [<6–28.5]	0.060
Striatal DAT BP_ND_	4.47 ± 1.31	4.87 ± 1.22	0.501
Hypothalamic SERT BP_ND_	0.32 [0.18–0.53]	0.45 [0.33–0.71]	0.248
Thalamic SERT BP_ND_	0.60 [0.55–0.69]	0.53 [0.36–0.62]	0.165

Data are mean ± SD or median [IQR] and were using *t*-tests or Mann-Whitney *U* tests.

**Table 2:** The effects of a 24-h fast on metabolic and neuroimaging parameters, as compared with a 12-h fast.

	Lean men (n = 10)	Men with obesity (n = 9)	p^b^
REE (%)	9.0 ± 16.5	−3.4 ± 7.0	0.054
RQ (%)	−10.9 [−20.3 to −2.7]^a^	0.0 [−13.3–6.0]	0.063
Fasting glucose (%)	−9.7 ± 7.9^a^	−6.7 ± 5.6^a^	0.360
Fasting FFA (%)	80.4 ± 101.2	26.6 ± 40.4	0.155
Fasting insulin (%)	−40.3 [−58.6 to −19.2]^a^	−28.6 [−33.2–9.8]	0.133
Fasting glucagon (%)	3.3 ± 7.6	16.3 ± 21.4	0.088
Fasting leptin (%)	−40.9 ± 18.6^a^	−22.0 ± 21.7^a^	0.056
Fasting ghrelin (%)	−29.2 [−47.3–13.1]	0.0 [−3.3–8.3]	0.156
Striatal DAT BP_ND_ (%)	4.3 [−4.9–21.3]	−9.9 [−23.0–20.3]	0.327
Hypothalamic SERT BP_ND_ (%)	48.3 ± 76.4^a^	−36.3 ± 71.1	0.029
Thalamic SERT BP_ND_ (%)	−8.9 ± 28.1	−2.0 ± 44.0	0.685

Data are mean ± SD or median [IQR] and expressed as percentage difference between the 12-h and 24-h fasting interventions.

a p < 0.05 for within-group difference on paired *t*-test or Wilcoxon signed rank test.

b For lean men vs men with obesity on t-test or Mann Whitney U test.

## Abbreviations:

^123^I-FP-CIT: ^123^I-*N*-ω-fluoropropyl-2β-carboxymethoxy-3β-(4-iodophenyl)nortropane
ANOVA: analysis of variance
BMI: body mass index
BP_ND_: binding potential
DAT: dopamine transporter
FFAR: free fatty acid receptor
FFA: free fatty acid
GPCR: G-protein-coupled receptor
IQR: interquartile range
MRI: magnetic resonance imaging
REE: resting energy expenditure
ROI: region-of-interest
RQ: respiratory quotient
SD: standard deviation
SERT: serotonin transporter
SPECT: single-photon emission computed tomograph
T1w: T1-weighted
