# Supplementary material for: The response to prolonged fasting in hypothalamic serotonin transporter availability is blunted in obesity
Source: Metabolism. Author manuscript; Available in PMC 2022 Apr 9. (PMC8994212; doi:10.1016/j.metabol.2021.154839)
Supplement: SUPPLEMENTARY TABLES S1 andS2 [file NIHMS1787219-supplement-SUPPLEMENTARY_TABLES_S1_andS2.doc]

#### Supplementary materials

| **Supplemental Table S1.** C**haracteristics of insulin-sensitive and -resistant individuals after the 12-hour fast.** | | | |
| --- | --- | --- | --- |
|  | **Insulin-sensitive men (n=13)** | **Insulin-resistant men**  **(n=6)** | **P^a^** |
| **Age (years)** | 64±7 | 65±9 | 0.713 |
| **Length (cm)** | 175±8 | 177±7 | 0.600 |
| **Weight (kg)** | 73 [63-96] | 97 [93-109] | 0.035 |
| **BMI (kg/m^2^)** | 24.3 [22.1-28.0] | 31.7 [30.3-34.3] | 0.014 |
| **Waist circumference (cm)** | 91 [88-104] | 115 [108-127] | 0.014 |
| **REE (kcal/kg/day)** | 19.4±3.7 | 19.5±2.3 | 0.945 |
| **RQ** | 0.78±0.06 | 0.75±0.05 | 0.214 |
| **Glucose (mmol/L)** | 4.9±0.4 | 5.8±1.7 | 0.261 |
| **FFA (mmol/L)** | 0.49 [0.39-0.58] | 0.41 [0.33-0.67] | 0.568 |
| **Insulin (pmol/L)** | 36 [26-58] | 155 [120-183] | 0.001 |
| **Glucagon (ng/L)** | 83 [65-115] | 121 [77-136] | 0.087 |
| **Leptin (μg/L)** | 6 [5-11] | 25 [19-40] | 0.004 |
| **Ghrelin (pg/mL)** | 30.2 [18.9-47.4] | 6.9 [<6-27.2] | 0.028 |
| **Hypothalamic SERT BP_ND_** | 0.39±0.20 | 0.49±0.26 | 0.368 |
| **Thalamic SERT BP_ND_** | 0.56±0.15 | 0.54±0.18 | 0.814 |
| **Striatal DAT BP_ND_** | 4.75±1.34 | 4.47±1.31 | 0.663 |
| Data are mean ± SD or median [IQR].  **^a^** for insulin-sensitive vs insulin-resistant on t-test or Mann Whitney U test. | | | |

| **Supplemental Table S2. Multiple regression analysis for fasting-induced changes in striatal DAT availability.** | | | | | | |
| --- | --- | --- | --- | --- | --- | --- |
|  | **B** | **95% CI for B** | **SE_B_** | **β** | **R^2^** | **Adjusted R^2^** |
| **Model** |  |  |  |  | 0.535* | 0.441* |
| **Intercept** | 4.288 | -9.680 – 18.256 | 6.269 |  |  |  |
| **Change in plasma FFA (%)** | -0.122* | -0.235 – -0.009 | 0.051 | -0.520* |  |  |
| **Change in plasma insulin (%)** | -0.280* | -0.542 – -0.019 | 0.117 | -0.516* |  |  |
| B, unstandardized regression coefficient; CI, confidence interval; SE_B_, Standard error of the coefficient; β, standardized coefficient; R^2^, coefficient of determination. * p <0.05 | | | | | | |
